# Identification of fibrosis in hypertrophic cardiomyopathy: a radiomic study on cardiac magnetic resonance cine imaging

**DOI:** 10.1007/s00330-022-09217-0

**Published:** 2022-11-05

**Authors:** Cailing Pu, Xi Hu, Sangying Lv, Yan Wu, Feidan Yu, Wenchao Zhu, Lingjie Zhang, Jingle Fei, Chengbin He, Xiaoli Ling, Fuyan Wang, Hongjie Hu

**Affiliations:** 1 Department of Radiology, Sir Run Run Shaw Hospital, Zhejiang University School of Medicine, No.3 Qingchun East Road, Hangzhou, 310016 Zhejiang Province China; 2 Department of Radiology, Shaoxing People’s Hospital, Shaoxing, Zhejiang Province China; 3 Department of Radiology, Lishui Municipal Central Hospital, Lishui, Zhejiang Province China; 4 Department of Radiology, The Affiliated Hospital of Hangzhou Normal University, Hangzhou, Zhejiang Province China

**Keywords:** Cardiomyopathy, Hypertrophic, Fibrosis, Magnetic resonance imaging, Machine learning

## Abstract

**Objectives:**

Hypertrophic cardiomyopathy (HCM) often requires repeated enhanced cardiac magnetic resonance (CMR) imaging to detect fibrosis. We aimed to develop a practical model based on cine imaging to help identify patients with high risk of fibrosis and screen out patients without fibrosis to avoid unnecessary injection of contrast.

**Methods:**

A total of 273 patients with HCM were divided into training and test sets at a ratio of 7:3. Logistic regression analysis was used to find predictive image features to construct CMR model. Radiomic features were derived from the maximal wall thickness (MWT) slice and entire left ventricular (LV) myocardium. Extreme gradient boosting was used to build radiomic models. Integrated models were established by fusing image features and radiomic models. The model performance was validated in the test set and assessed by ROC and calibration curve and decision curve analysis (DCA).

**Results:**

We established five prediction models, including CMR, R1 (based on the MWT slice), R2 (based on the entire LV myocardium), and two integrated models (I_CMR+R1_ and I_CMR+R2_). In the test set, I_CMR+R2_ model had an excellent AUC value (0.898), diagnostic accuracy (89.02%), sensitivity (92.54%), and F1 score (93.23%) in identifying patients with positive late gadolinium enhancement. The calibration plots and DCA indicated that I_CMR+R2_ model was well-calibrated and presented a better net benefit than other models.

**Conclusions:**

A predictive model that fused image and radiomic features from the entire LV myocardium had good diagnostic performance, robustness, and clinical utility.

**Key Points:**

*• Hypertrophic cardiomyopathy is prone to fibrosis, requiring patients to undergo repeated enhanced cardiac magnetic resonance imaging to detect fibrosis over their lifetime follow-up.*

*• A predictive model based on the entire left ventricular myocardium outperformed a model based on a slice of the maximal wall thickness.*

*• A predictive model that fused image and radiomic features from the entire left ventricular myocardium had excellent diagnostic performance, robustness, and clinical utility.*

**Supplementary Information:**

The online version contains supplementary material available at 10.1007/s00330-022-09217-0.

## Introduction

Myocardial fibrosis is a characteristic pathological manifestation of hypertrophic cardiomyopathy (HCM). Myocardial fibrosis in patients with HCM is a predictor of poor prognosis, including sudden cardiac death, heart failure, and ventricular arrhythmia [1, 2]. Cardiac magnetic resonance (CMR) is the gold standard non-invasive modality for cardiac structure and function assessment. Late gadolinium enhancement (LGE) sequence can detect fibrosis and quantify scarred myocardium through post-processing techniques [3]. However, patients with HCM are often younger than the general population undergoing enhanced CMR scanning and need multiple CMRs to assess lifetime disease progression, nearly 1/3–1/2 of these patients are estimated to have no fibrosis [2, 4, 5]. Several studies have shown that gadolinium should be used with caution in patients with chronic kidney disease and is contraindicated in those with gadolinium allergy. Repeated gadolinium injections might result in its deposition in the central nervous system [6, 7]. Therefore, it is important to find a personalized strategy to identify patients with HCM who require LGE scanning. While patients at high risk of fibrosis should undergo LGE for further quantitative and prognostic analysis, exposure to a contrast agent could be minimized in those at low risk of fibrosis, reducing scan time and healthcare costs.

The application and exploration of extensive data mining in clinical medicine have vastly grown in recent years. Radiomics has become increasingly widespread in heart diseases as it can characterize the myocardial tissue [8–11]. Neisius et al demonstrated that radiomic analysis of native T1 mapping could outperform global native T1 values in discriminating between HCM and hypertensive heart disease as well as identifying LGE(−) patients for avoiding gadolinium administration [12, 13]. However, unlike cine sequence, mapping technology is unavailable in most hospitals. Schofield et al analyzed texture features of the mid-left ventricular slice in cine imaging, showing that radiomics could distinguish the various left ventricular (LV) hypertrophy etiologies [14]. Nevertheless, a comparison between radiomics studies based on the entire LV myocardium and a LV myocardial slice has not been reported.

The study aimed to establish models that fuse image features and texture characteristics based on cine imaging to detect fibrosis in patients with HCM and compare the performance of models constructed using a single slice and the entire LV myocardium.

## Material and methods

### Study population

**Fig. 1:**
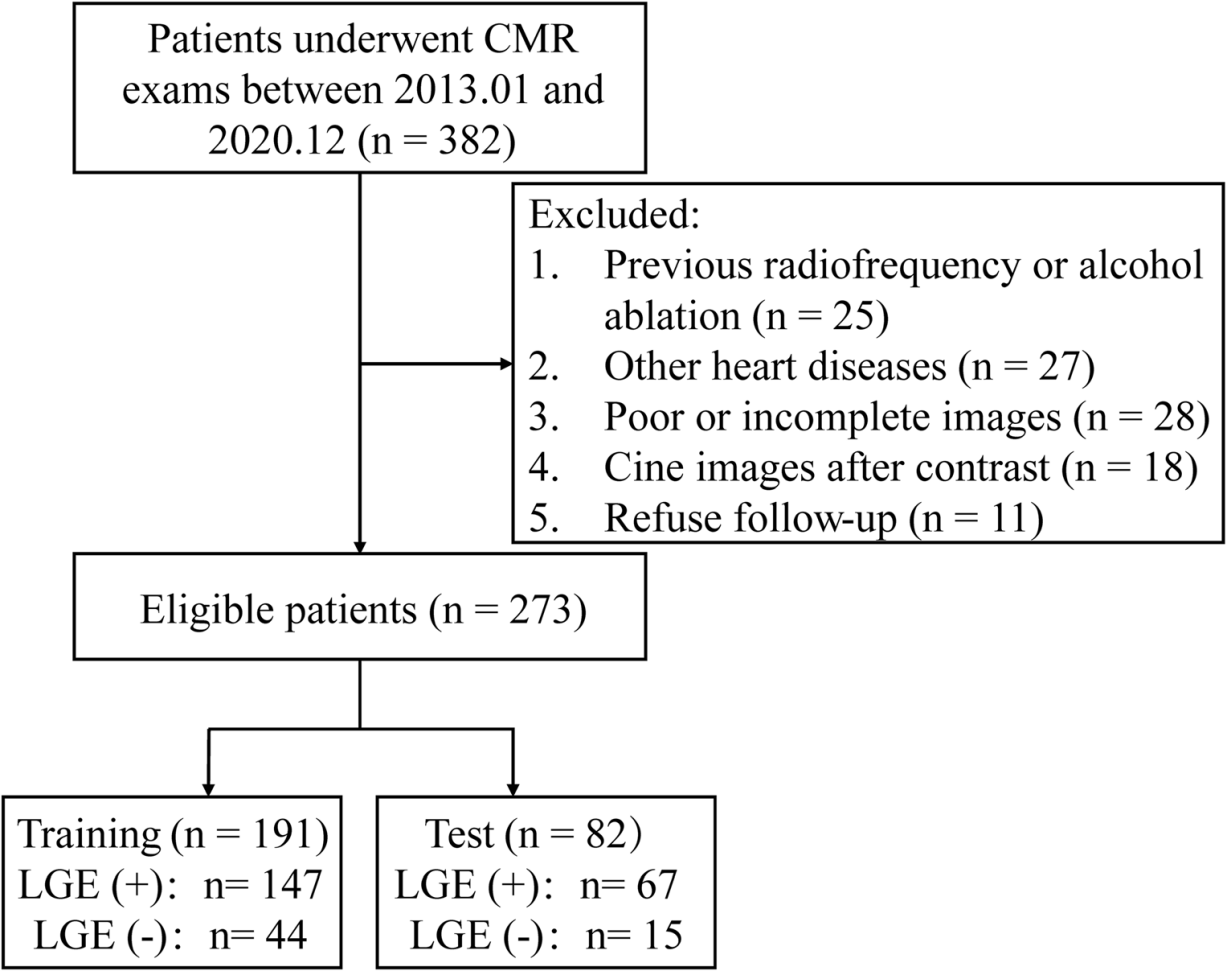
Flowchart of patient selection in this study

The ethics committee of our hospital approved this study and waived the requirement for informed consent due to its retrospective nature. The study included patients with HCM who underwent CMR scans between January 2013 and December 2020 (Fig. 1). HCM was defined referred to previous guideline: maximal LV thickness on CMR images ≥ 15 mm or ≥ 13 mm with a documented family history of HCM [2]. The exclusion criteria were as follows: poor or incomplete images, previous radiofrequency or alcohol ablation, myocardial infarction, and myocardial hypertrophy due to other causes such as hypertensive cardiomyopathy, poorly controlled hypertension, cardiac amyloidosis, valvular disease, and Fabry disease.

### Cardiac magnetic resonance imaging

CMR scans were performed on 1.5-T magnetic resonance scanners (GE Signa Excite HD or Siemens MAGNETOM Avanto) with a phased-array body coil. Short-axis cine images covering the entire LV and long-axis cine images (two/ three/ four-chamber) were acquired using a retrospective electrocardiogram-gated and balanced steady-state free precession sequence. The respective parameters were as follows: slice thickness/gap, 8 mm/2 mm; repetition time (TR), 3.5 and 2.64 ms; echo time (TE), 1.5 and 1.11 ms; flip angle, 45° and 56°; field of view (FOV), 360 × 360 and 340 × 276 mm^2^; acquired matrix, 224 × 224 and 192 × 125 pixels^2^; reconstructed matrix, 512 × 512 and 192 × 156 pixels^2^; temporal resolution, 49 and 47.52 ms. The array spatial and sensitivity encoding technique (acceleration factor = 2) was used in the GE scanner, while the generalized auto-calibrating partially parallel acquisition (acceleration factor = 2) in the Siemens scanner. LGE images were obtained 8–10 min after intravenous injection of 0.15–0.2 mmol/kg of gadopentetate dimeglumine (Beilu) with the following parameters: slice thickness/gap, 8 mm/2 mm; TR, 6.6 and 2.5 ms; TE, 3.1 and 1.09 ms; flip angle, 20° and 40°; FOV, 360 × 324 and 340 × 255 mm^2^ (Supplementary Table S1).

### Imaging analysis

Cardiac function and strain analysis were performed using CVI42, Version 5.13.5 (Circle Cardiovascular Imaging Inc.). LV function, including LV ejection fraction (LVEF), LV end-diastolic volume index (LVEDVI), LV end-systolic volume index (LVESVI), systolic volume index, and LV mass index (LVMI), was calculated automatically by loading short-axis images into the short-3D module. A stack of long- (two-/three-/four-chamber) and short-axis images were loaded into the tissue tracking module for two-dimensional strain analysis. Endocardial and epicardial borders at end-diastolic LV were automatically drawn to track the myocardium throughout the heart cycle and manually adjusted when necessary for optimal tracking. Then, peak values of myocardial strains and strain rates were derived: global radial, circumferential, and longitudinal strains (GRS, GCS, GLS); global radial, circumferential, and longitudinal strain rates (GRSr, GCSr, GLSr); early global radial, circumferential, and longitudinal strain of diastolic rates (GRSDr, GCSDr, GLSDr). The end-diastolic maximum wall thickness (MWT) was measured in the short-axis images. Left atrial anteroposterior diameter was measured on a three-chamber view.

LGE analysis was obtained in the tissue characteristic module. Radiologist A, with 4 years of CMR experience, visually estimated LGE in short- and long-axis views. Patients were classified as LGE(+) if they had myocardial enhancement in at least one segment. If uncertain, a senior radiologist was consulted, reaching a consensus through discussion. LGE extent was quantified as the percentage of total LV mass (%LGE) using a grayscale threshold of > 5 SDs from remote normal myocardium. The HCM cohort was divided into LGE (+) and LGE (−) groups.

### Radiomic features extraction

Radiologist A manually delineated the entire LV myocardium at the end of diastole in short-axis cine views as the region of interest using ITK-SNAP, version 3.8.0 (http://www.itksnap.org/). Before feature extraction, cine images were interpolated to voxel sizes of 1 × 1 × 4 mm^3^ using a SimpleITK nearest-neighbor resampling filter to reduce within-image variability. A min-max normalization was performed for all images to map the pixel intensity to 0–255. The Pyradiomics package, version 3.0.1 (https://github.com/Radiomics/pyradiomics), extracted 1130 and 1688 features from the MWT slice and the entire LV myocardium, respectively, including four categories: 18 first-order, 14 shape (2D/3D), and 75 texture features for both, and 1023 and 1581 high-order features, respectively.

### Radiomic features selection

Radiologist A delineated images from 30 randomly selected patients with HCM twice one month apart to test intraclass reproducibility. The 30 patients were also assessed by radiologist B, who had 5 years of experience in CMR, to assess interclass reproducibility. Features with intra- and interclass correlation coefficient (ICC) > 0.85 were considered reproducible and retained for further analysis. Subsequently, the Boruta algorithm reduced the dimensionality of the total radiomic features [15]. The importance of each feature was calculated and ranked and the top 100 features were retained. Extreme gradient boosting (XGBoost) was selected as the machine learning technique for prediction.

### Model construction and validation

**Fig. 2:**
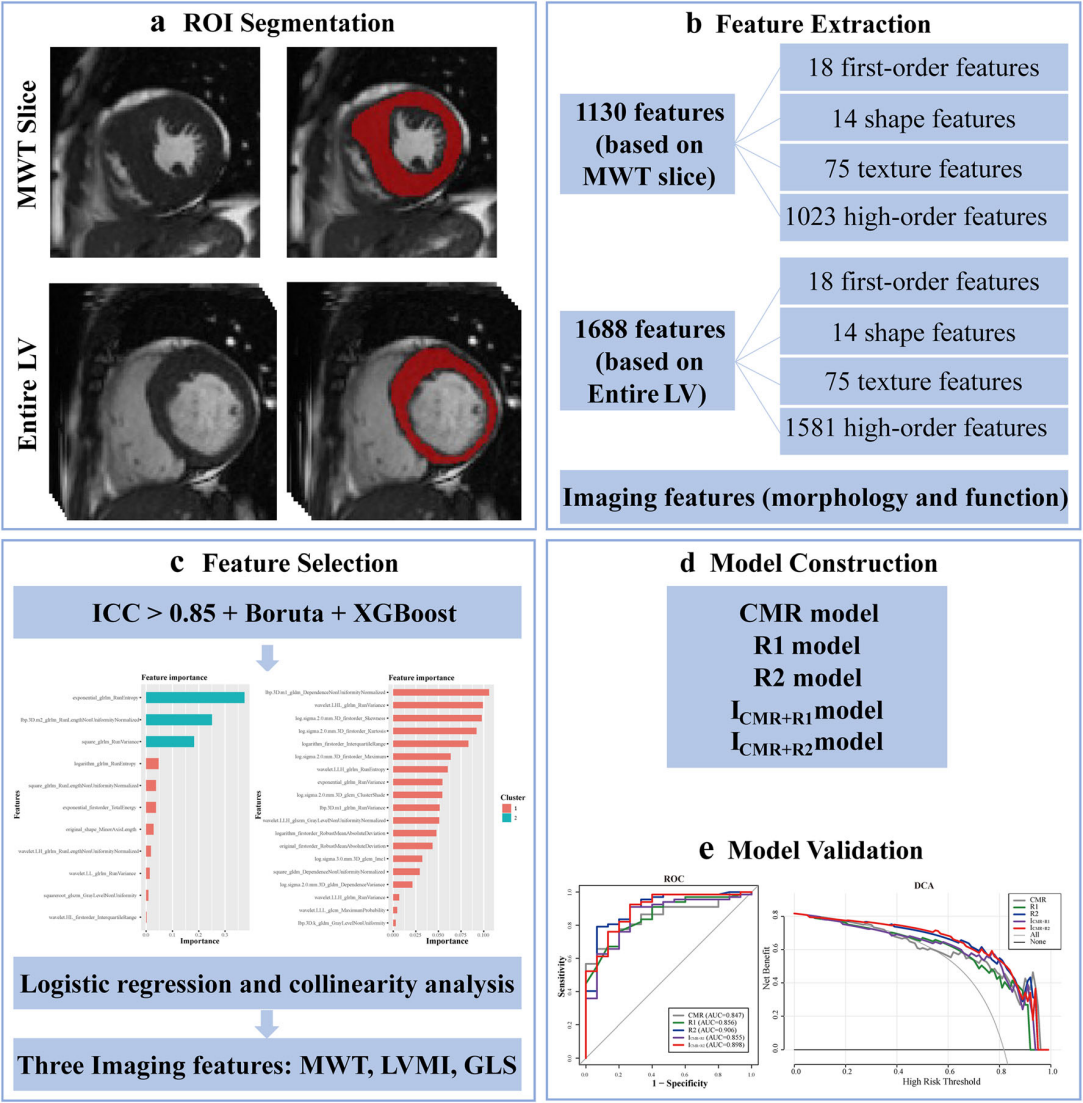
Radiomics workflow in this study

CMR imaging parameters with significant differences were selected in the training set using logistic regression and collinearity analysis. These were used to build the CMR model. R1 and R2 models were constructed based on the MWT slice and entire LV myocardium, respectively, using radiomic features selected by XGBoost. I_CMR+R1_ and I_CMR+R2_ models were built by integrating CMR model and R1 or R2 model through logistic regression. Receiver operating characteristic (ROC) curves evaluated the models’ diagnostic performance. Calibration curve and Hosmer-Lemeshow test evaluated the calibration performance and goodness of fit. Decision curve analysis (DCA) assessed the net benefits of models. Net reclassification improvement (NRI) and integrated discrimination improvement (IDI) evaluated the incremental value of I_CMR+R2_ for identifying fibrosis. Figure 2 shows the study methodology workflow.

### Statistical analysis

Data analysis was performed using IBM SPSS Statistics for Windows, version 23.0 (IBM Corp.) and R studio (version 4.1.2, http://www.r-project.org/). Continuous variables were expressed as mean ± SD or median (interquartile range). Shapiro Wilk test evaluated the normality of data distributions. Student’s *t*-test or Mann–Whitney test was used appropriately to assess differences between groups. Categorical data were expressed as frequencies (percentages), and the chi-square test or Fisher’s exact test compared differences between groups. Statistical significance was set at *p* < 0.05.

## Results

### Clinical data and CMR variables of HCM patients

This study included 273 patients with HCM (training set, 70%; test set, 30%). Patients and CMR characteristics are summarized in Table 1. In the training set, the median age was 55 years, 67.54% were male, and 147 (76.96%) were LGE(+). More patients had a history of non-sustained ventricular tachycardia and LVEF < 50% in the LGE(+) than in the LGE(–) group, showing higher MWT and LVMI and lower strains, including GRS, GCS, GLS, GRSr, GCSr, GLSr, GRSDr, and GLSDr (all *p* < 0.05; Table 1). In the test set, the median age was 53.5 years, most were male (73.17%) and 67 (81.70%) were LGE(+). LVEDVI, LVESVI, MWT, LVMI, GRS, GCS, GLS, GLSr, and GLSDr were significantly different between LGE(–) and LGE(+) groups (all *p* < 0.05; Table 1). The groups were similar for all other variables.

**Table 1:** Clinical characteristics and CMR parameters of HCM patients in the training and test sets

Variable	Training set (191)			Test set (82)			*p*^†^
	LGE(−) (44)	LGE(+) (147)	*p*	LGE(−) (15)	LGE(+) (67)	*p*	
Clinical data							
Age (years)	54.5 (48.75, 63.25)	56 (47, 63)	0.812	55 (47, 61.5)	53 (45.5, 60)	0.623	0.129
Gender, M	33 (75)	96 (65.31)	0.307	10 (66.67)	50 (74.63)	0.759	0.435
Height (cm)	170 (161.5, 174)	166 (160, 172)	0.149	165.4 ± 8.48	166.42 ± 7.57	0.646	0.947
Weight (kg)	72.77 ± 12.86	68.21 ± 12.02	0.031*	72 (67, 76)	71 (61, 79)	0.787	0.282
BSA (m^2^)	1.84 ± 0.20	1.77 ± 0.18	0.031*	1.84 (1.73, 1.88)	1.79 (1.66, 1.92)	0.971	0.366
Hypertension (*n*, %)	17 (38.64)	60 (40.82)	0.934	6 (40)	24 (35.82)	0.994	0.658
DM (*n*, %)	2 (4.55)	10 (6.8)	0.737	2 (13.33)	7 (10.45)	0.666	0.277
Family history (*n*, %)	4 (9.09)	27 (18.37)	0.168	1 (6.67)	11 (16.42)	0.451	0.880
History of SCD (*n*, %)	2 (4.55)	9 (6.12)	1.000	0 (0)	5 (7.46)	0.579	1.000
Syncope (*n*, %)	4 (9.09)	26 (17.69)	0.238	2 (13.33)	9 (13.43)	1.000	0.763
Aneurysm (*n*, %)	0 (0)	6 (4.08)	0.339	0 (0)	1 (1.49)	1.000	0.678
NSVT^a^ (*n*, %)	2 (7.41)	33 (29.73)	0.015*	0 (0)	15 (27.27)	0.057	0.814
CMR data							
Scanner			0.835			0.631	0.715
GE, *n* (%)	28 (14.66)	91 (47.64)		11 (13.41)	42 (51.22)		
Siemens, *n* (%)	16 (8.38)	56 (29.32)		4 (4.88)	25 (30.49)		
LVEF (%)	66.88 (62.78, 73.18)	65.89 (58.55, 70.27)	0.076	68.27 (62.49, 72.47)	65.78 (61.39, 69.21)	0.262	0.836
LVEF < 50% (*n*, %)	0 (0)	15 (10.2)	0.024*	0 (0)	6 (8.96)	0.586	1.000
LVEDVI (mL/m^2^)	78.28 (67.46, 85.88)	75.99 (69.34, 90.60)	0.734	72.21 (65, 79.92)	80.83 (73.06, 93.36)	0.017*	0.286
LVESVI (mL/m^2^)	24.86 (21.09, 29.11)	25.69 (22.11, 35.40)	0.152	22.46 (21.14, 26.73)	27.18 (22.84, 32.98)	0.033*	0.597
SVI (mL/m^2^)	52.98 ± 9.48	49.88 ± 10.22	0.074	49.9 ± 13.6	52.67 ± 9.7	0.359	0.246
LAD-AP (mm)	39.00 (34, 43)	40 (36, 44)	0.198	38.93 ± 7.32	42.06 ± 6.63	0.109	0.158
MWT (mm)	16.5 (15, 20)	21 (18, 25)	< 0.001*	17 (15.5, 18)	22 (19.5, 27)	< 0.001*	0.247
LVMI (g/m^2^)	65.64 (57.43, 74.40)	72.58 (59.82, 90.63)	0.018*	62.88 (54.72, 67.35)	81.56 (67.58, 98.62)	0.017*	0.021*
LGE (*n*, %)	0	147 (76.56)	–	0	67 (81.71)	–	0.476
LGE mass^b^ (g)	0	7.18 (3.19, 16.70)	< 0.001*	0	5.64 (2.82, 20.34)	< 0.001*	0.691
%LGE^b^	0	5.40 (2.46, 11.28)	< 0.001*	0	4.54 (2.29, 10.75)	< 0.001*	0.937
GRS (%)	25.32 ± 6.44	22.1 ± 7.81	0.013*	26.44 (21.37, 31.86)	19.51 (15.67, 25.68)	0.026*	0.314
GCS (%)	**−**15.21 ± 3.07	**−**13.48 ± 3.82	0.007*	**−**15.74 ± 4.07	**−**13.01 ± 3.80	0.015*	0.463
GLS (%)	**−**12.40 ± 2.96	**−**9.62 ± 3.21	< 0.001*	**−**12.72 (**−**14.32, **−**9.79)	**−**8.47 (**−**10.04, **−**6.14)	< 0.001*	0.006*
GRSr (1/s)	1.49 (1.21, 1.70)	1.27 (1.04, 1.62)	0.012*	1.43 ± 0.45	1.25 ± 0.38	0.123	0.195
GCSr (1/s)	**−**0.93 (**−**1.04, **−**0.84)	**−**0.85 (**−**0.99, **−**0.70)	0.011*	**−**0.86 ± 0.23	**−**0.81 ± 0.19	0.340	0.141
GLSr (1/s)	**−**0.74 (**−**0.90, **−**0.65)	**−**0.59 (**−**0.73, **−**0.46)	< 0.001*	**−**0.68 (**−**0.85, **−**0.59)	**−**0.53 (**−**0.68, **−**0.44)	0.002*	0.014*
GRSDr (1/s)	**−**1.06 (**−**1.29, **−**0.77)	**−**0.86 (**−**1.21, **−**0.68)	0.031*	**−**1.10 (**−**1.42, **−**0.88)	**−**0.84 (**−**1.23, **−**0.56)	0.084	0.881
GCSDr (1/s)	0.61 ± 0.15	0.56 ± 0.16	0.057	0.66 (0.54, 0.73)	0.57 (0.44, 0.70)	0.259	0.527
GLSDr (1/s)	0.58 (0.54, 0.72)	0.48 (0.39, 0.58)	< 0.001*	0.53 (0.45, 0.70)	0.45 (0.36, 0.54)	0.031*	0.020*

Data are presented as mean ± SD or median (interquartile range) or absolute number (percentage)

*Abbreviations*: *BSA* body mass index, *CMR* cardiac magnetic resonance, *DM* diabetes mellitus, *GCS(r)* global circumferential strain (rate), *GCSDr* global circumferential strain of diastolic rate, *GLS(r)* global longitudinal strain (rate), *GLSDr* global longitudinal strain of diastolic rate, *GRS(r)* global radial strain (rate), *GRSDr* global radial strain of diastolic rate, *HCM* hypertrophic cardiomyopathy, *LAD-AP* left atrial anteroposterior diameter, *LGE* late gadolinium enhancement, *LVEDVI* left ventricular end-diastolic volume index, *LVEF* left ventricular ejection fraction, *LVESVI* left ventricular end-systolic volume index, *LVMI* left ventricular mass index, *MWT* maximum wall thickness, *NSVT* non-sustained ventricular tachycardia, *SCD* sudden cardiac death, *SVI* stroke volume index

^†^Comparison between the training and test sets

*p < 0.05

^a b^Not included all HCM patients

### Radiomic feature selection

The radiomics workflow is illustrated in Fig. 2. A total of 1688 and 1130 features were extracted from the entire LV myocardium and MWT slice respectively. Among them, 1041 and 546 features had good repeatability with ICC > 0.85. The XGBoost algorithm selected the most important and robust features. Supplementary Figure S1 depicts the importance level of selected features from the MWT slice and entire LV myocardium. The predictive score was much higher in the LGE(+) group than in the LGE(–) group (Supplementary Figure S2). The top three radiomic features in R1, the top five radiomic features in R2, and correlated interpretations in LGE(+) with patients are shown in Supplementary Table S2.

### Model construction and evaluation

**Fig. 3:**
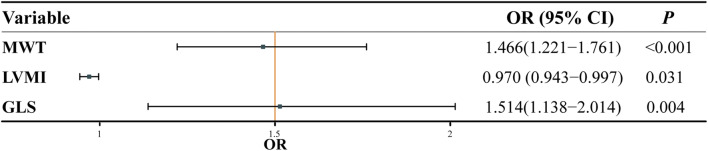
Multivariate logistic regression analysis indicated maximum wall thickness (MWT), left ventricular mass index (LVMI), and global longitudinal strain (GLS) as independent predictors of LGE(+) patients

**Fig. 4:**
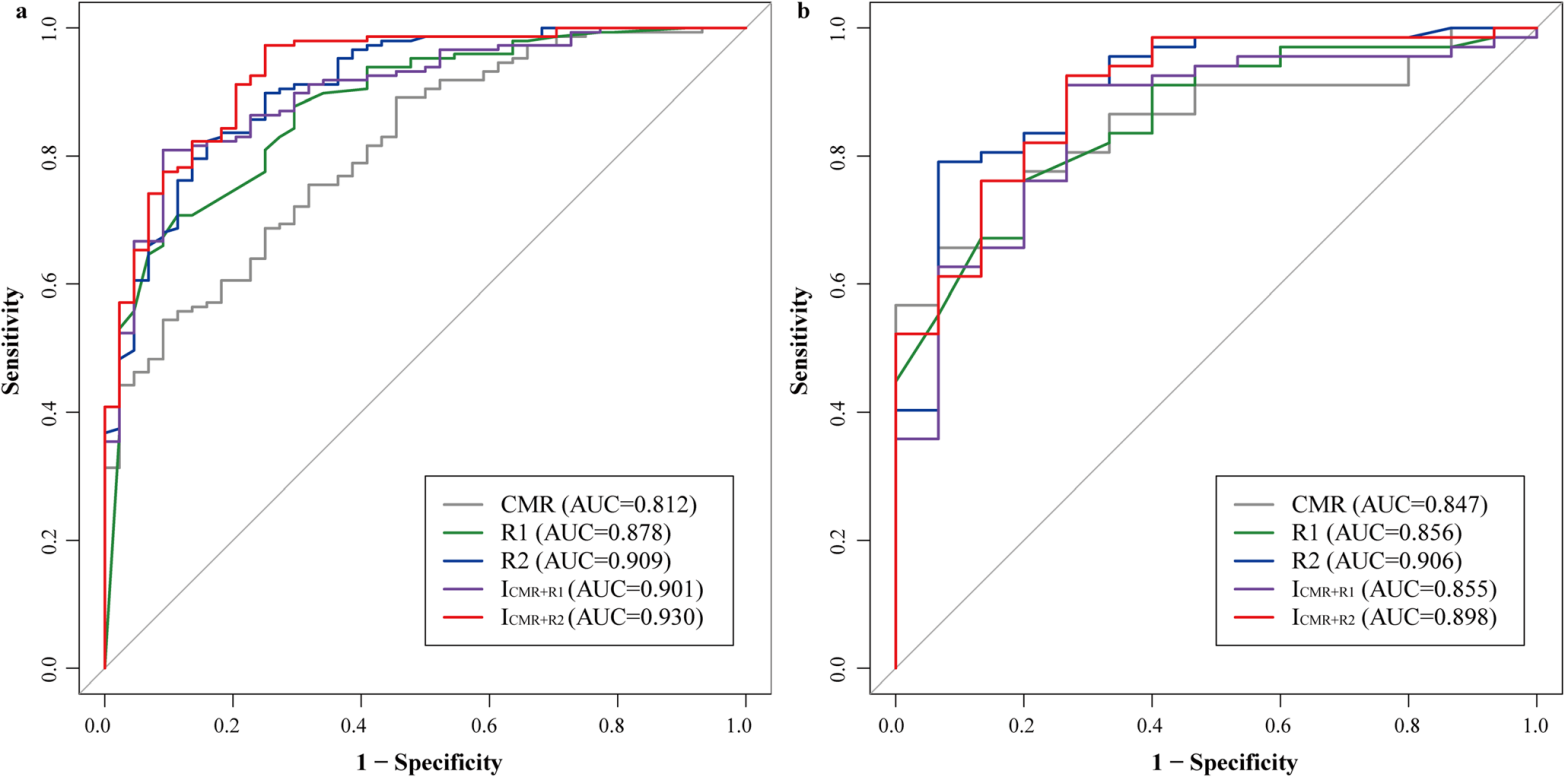
Receiver operating characteristic curves of five models for identifying LGE(+) patients in the training (**a**) and test (**b**) sets

**Fig. 5:**
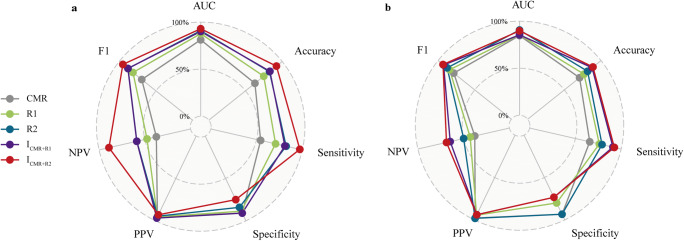
Radar plots showing the diagnostic efficacy of five models in the training (**a**) and test (**b**) sets

Univariate and multivariate logistic regression and collinearity analyses revealed that MWT, LVMI, and GLS were independent predictors of LGE(+) (Fig. 3). Therefore, we established five prediction models: the CMR model, two radiomic models (R1 and R2), and two integrated models (I_CMR+R1_ and I_CMR+R2_). In the training set, R2, I_CMR+R1_, and I_CMR+R2_ models could better distinguish between LGE(+) and LGE(–) groups (AUC values were 0.909, 0.901, and 0.930, respectively) than CMR and R1 models (Fig. 4a). In the test set, R2 and I_CMR+R2_ models showed an excellent predictive ability for LGE(+) patients (AUC values were 0.906 and 0.898, respectively; Fig. 4b). The radar plots (Fig. 5) and Table 2 show that the I_CMR+R2_ model had high diagnostic accuracy, sensitivity, NPV, and F1 score for differentiating LGE(+) from LGE(–) patients in the training set (92.15%, 97.28%, 89.19%, and 95.02%, respectively) and test set (89.02%, 92.54%, 68.75%, and 93.23%, respectively). The R2 model achieved a specificity of 93.33% in the test cohort. Supplementary Figure S3 illustrates myocardial appearance in cine imaging by radiomic analysis to reveal fibrosis.

**Table 2:** Diagnostic performance of CMR model, radiomic models, and integrated models in the training and test sets

Model	Training set (*n* = 191)							Test set (*n* = 82)						
	AUC (95% CI)	Accuracy	Sensitivity	Specificity	PPV	NPV	F1 score	AUC (95% CI)	Accuracy	Sensitivity	Specificity	PPV	NPV	F1 score
**CMR**	0.812 (0.750–0.865)	62.83%	54.42%	90.91%	95.24%	37.38%	69.26%	0.847 (0.750–0.917)	70.73%	65.67%	93.33%	97.78%	37.84%	78.57%
R1	0.878 (0.823–0.921)	74.87%	70.75%	88.64%	95.41%	47.56%	81.25%	0.856 (0.761–0.924)	76.83%	76.12%	80.00%	94.44%	42.86%	84.30%
R2	0.909 (0.859–0.946)	82.72%	82.31%	84.09%	94.53%	58.73%	88.00%	0.906 (0.821–0.959)	81.71%	79.10%	93.33%	98.15%	50.00%	87.60%
I_CMR+R1_	0.901 (0.850–0.940)	83.25%	80.95%	90.91%	96.75%	58.82%	88.15%	0.855 (0.760–0.923)	87.80%	91.04%	73.33%	93.85%	64.71%	92.42%
I_CMR+R2_	0.930 (0.884–0.962)	92.15%	97.28%	75.00%	92.86%	89.19%	95.02%	0.898 (0.811–0.954)	89.02%	92.54%	73.33%	93.94%	68.75%	93.23%

*AUC* area under the curve, *PPV* positive predictive value, *NPV* negative predictive value; others, see Table 1

**Fig. 6:**
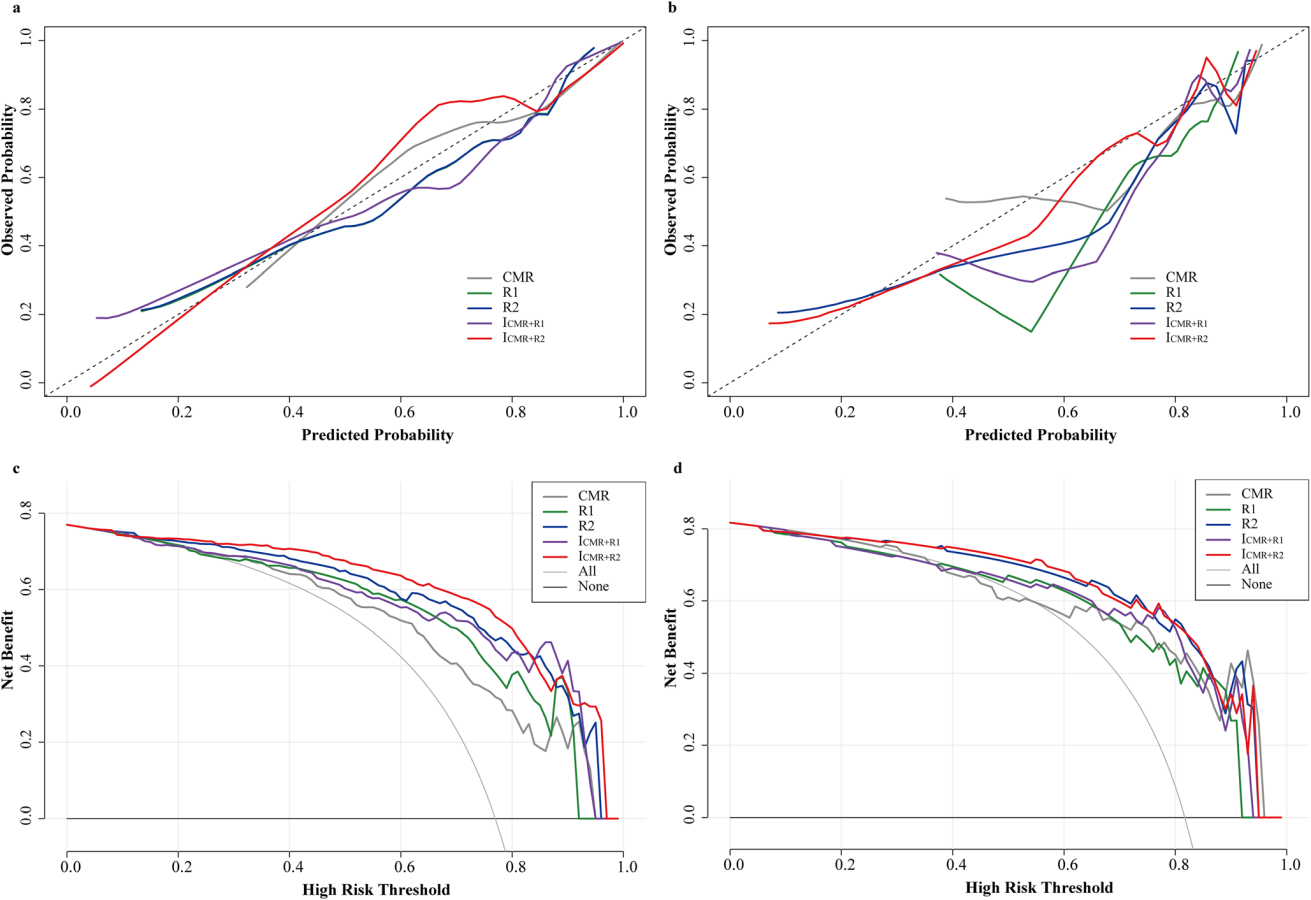
Calibration curves and decision curve analyses of the various models (**a**, **c**: training set; **b**, **d**: test set). Calibration curves depict the calibration performance of models in terms of an agreement between predicted and observed LGE(+). The closer the curve is to the dotted black line, the better the model’s predictive accuracy is. For decision curves, the vertical axis measures standardized net benefit, while the horizontal axis shows the corresponding risk threshold. The decision curves show that performing unenhanced CMR based on the indications of R2 and I_CMR+R2_ models had better net benefits than other models for threshold probabilities ranging from 40 to 90%

Furthermore, we evaluated the calibration of the five models and their impact on clinical decision-making. The calibration plots indicated that I_CMR+R2_ model was well calibrated, with a mean predicted probability for each group close to the observed probability (Fig. 6a, b). This was further proved by the Hosmer-Lemeshow test with *p* values > 0.05 in both cohorts, suggesting no reason to reject the null hypothesis of no difference between the predicted and observed LGE(+) probability in each group. The DCA results comparing the performance of five models are shown in Fig. 6c and d. The results suggested the use of unenhanced CMR based on the indications of R2 and I_CMR+R2_ models had better net benefits than other models for threshold probabilities ranging from 40 to 90%. Furthermore, I_CMR+R2_ model yielded significantly positive NRI and IDI compared to the CMR model in the training (0.854 and 0.302, respectively) and test set (0.498 and 0.136, respectively), indicating that the I_CMR+R2_ model could improve the discrimination and reclassification for fibrosis in HCM. Moreover, we performed a subgroup analysis of R2 and I_CMR+R2_ models. AUC values in the GE and Siemens scanners were similar to the mixed data in both datasets (Supplementary Table S3), implying that our models worked equally well on data from the two scanners.

## Discussion

In this study, we constructed CMR, radiomics, and integrated models based on cine imaging to identify fibrosis in patients with HCM. The results in the independent test set showed that I_CMR+R2_ model could effectively detect 62 (92.54%) of the LGE(+) patients, while R2 model screened out 14 (93.33%) of LGE(–) patients. A model including radiomic features derived from the entire LV myocardium may provide a reliable and stable tool to identify patients with HCM at high risk of fibrosis and screen out those at low risk, avoiding unnecessary contrast-enhanced CMR scanning.

The apparent manifestation of HCM is myocardial thickening [1, 2]. LV wall thickness (MWT ≥ 15 mm) is one of the most important criteria for HCM diagnosis [2]. MWT is one of several clinical features used when recommending implantable cardioverter defibrillator implantation [16, 17]. Previous studies have demonstrated that myocardial hypertrophy and fibrosis can lead to reduced LV strain [18, 19]. This study found three imaging features independently associated with fibrosis. A CMR model including MWT, LVMI, and GLS had an AUC value of 0.847 to identify LGE(+) patients in the test cohort. However, the CMR model showed only mild accuracy (70.73%), sensitivity (65.67%), and F1 score (78.57%). In clinical practice, we often encounter situations where the LV wall is thickened but shows no LGE hyperintensity [20]. Thus, identifying fibrosis based on imaging features alone has a limited value.

Radiomics can conduct an in-depth analysis of medical images and extract a large number of features that the naked eyes cannot observe [8]. Baessler et al proved that radiomics of nonenhanced cine images could accurately diagnose subacute and chronic myocardial infarction [21]. Our study analyzed the radiomics of the MWT slice and entire LV myocardium. We found that almost all radiomic features in the R2 model were high-order features changed by various algorithms, which allowed identification of nonuniform and coarse myocardial texture associated with fibrosis. The results indicated that high-order features could provide rich and accurate differential diagnosis information [22, 23]. Among the five models, CMR, R1, and I_CMR+R1_ models had similar AUC values for identifying LGE(+) patients, suggesting that radiomic models based on a single myocardial slice have no advantage over imaging features in diagnosing fibrosis in HCM. In comparison, R2 and I_CMR+R2_ models had better discrimination power with AUC values of 0.906 and 0.898 in the test cohort. I_CMR+R2_ model correctly identified most LGE(+) patients (92.54%) who would benefit from LGE scanning. At least 93.33% of patients without fibrosis could avoid repeated enhanced CMR based on the R2 model. Therefore, after a comprehensive evaluation of the ROC curves, confusion matrix, decision curves, and reclassification indices, we believe that the I_CMR+R2_ model is the optimal tool to identify LGE(+) patients, while the R2 model is more suitable for screening out LGE(–) patients. In clinical practice, enhanced CMR is used most frequently to evaluate fibrosis. LGE scanning is recommended for patients with HCM without contraindications, regardless of the probability of fibrosis. This study proposed a personalized strategy, namely, performing gadolinium-free CMR in patients with HCM at low risk of fibrosis and LGE scanning in those at high risk of fibrosis. A multi-center study on 1099 patients with HCM established a deep learning model that combined morphology (MWT and radiomic features) and function (ventricular wall thickening) [24]. Among the 82 patients determined to have no fibrosis by the model in the validation set, 73 (89%) were true negative and could avoid enhanced CMR. Our study results show some differences. GLS, the final functional variable selected, was proved to be a relatively stable and convenient deformation factor [25, 26]. Although I_CMR+R2_ model could only identify 68.75% of patients without fibrosis, it still exhibited satisfactory discriminability and stability and excellent sensitivity (92.54%) and F1 score (93.23%), even with limited data.

Previous studies have shown that radiomics or deep learning based on CT and other MR sequences can identify patients with HCM at high risk of fibrosis. Qin et al [27] demonstrated that a radiomic model based on CT angiography achieved an AUC of 0.78 to assess the presence of fibrosis. However, CTA exposes patients to radiation and increases the risk of allergy to contrast agent. Zhang et al [28] proposed a deep learning method on cine images that could detect the presence, position, and size of chronic myocardial infarction. Zhang et al [29] showed that virtual native enhancement imaging could generate LGE-equivalent images using deep learning to fuse native T1 maps and cine images. Such images achieved high agreement with LGE in detecting the distribution and quantification of fibrosis. However, T1 mapping is only carried out in some large-scale tertiary hospitals, mostly for scientific research, with only a few centers adding mapping sequence to routine scans. Additionally, deep learning still has the issue of complex technology being regarded as a “black box.” Our I_CMR+R2_ model was constructed by radiomics based on routine cine images, presenting great discriminative performance and considerable clinical benefit, which would help promote its application in clinical practice.

This study had some limitations. First, it was a single-center study with a small sample size. A multi-center study with a larger sample is required to confirm the model effectiveness. Second, the results were based on two MR scanners. Future studies should test models for generality across diverse populations and scanners. Third, our study lacked a quantitative scar analysis. We are trying to collect multi-center data for further quantitative and prognostic studies, hoping to soon implement gadolinium-free scans in selected patients with HCM. Fourth, we assessed end-diastolic but not end-systolic cine images. This was because MWT data available for HCM diagnosis and CMR model training were acquired at the end of diastole. Finally, our study included slightly more patients with LGE(+) than in other studies, possibly because the patients referred to our center were screened by lower-level hospitals, resulting in a selection bias.

## Conclusion

A predictive model that fused radiomics from the entire LV myocardium had good diagnostic performance, robustness, and clinical utility. This model may represent an economical and feasible alternative follow-up plan for patients who do not have to undergo repeated enhanced CMR, avoiding unnecessary contrast injection.

## Supplementary Information

ESM 1 (DOCX 656 kb)

## Abbreviations

CMR: Cardiac magnetic resonance
GCS(r): Global circumferential strain (rate)
GCSDr: Global circumferential strain of diastolic rate
GLS(r): Global longitudinal strain (rate)
GLSDr: Global longitudinal strain of diastolic rate
GRS(r): Global radial strain (rate)
GRSDr: Global radial strain of diastolic rate
HCM: Hypertrophic cardiomyopathy
IDI: Integrated discrimination improvement
LGE: Late gadolinium enhancement
LV: Left ventricular
LVEDVI: Left ventricular end-diastolic volume index
LVEF: Left ventricular ejection fraction
LVESVI: Left ventricular end-systolic volume index
LVMI: Left ventricular mass index
MWT: Maximum wall thickness
NRI: Net reclassification improvement
XGBoost: Extreme gradient boosting

## Declarations

### Guarantor

The scientific guarantor of this publication is Prof. Hongjie Hu.

### Statistics and biometry

No complex statistical methods were necessary for this paper.

### Methodology

• retrospective

• diagnostic study

• performed at one institution

## References

[1] Baxi AJ, Restrepo CS, Vargas D, Marmol-Velez A, Ocazionez D, Murillo H (2016). Hypertrophic cardiomyopathy from a to Z: genetics, pathophysiology, imaging, and management. Radiographics.

[2] Ommen SR, Mital S, Burke MA (2020). 2020 AHA/ACC guideline for the diagnosis and treatment of patients with hypertrophic cardiomyopathy: a report of the American College of Cardiology/American Heart Association joint committee on clinical practice guidelines. J Am Coll Cardiol.

[3] Schulz-Menger J, Bluemke DA, Bremerich J (2020). Standardized image interpretation and post-processing in cardiovascular magnetic resonance - 2020 update : Society for Cardiovascular Magnetic Resonance (SCMR): Board of Trustees Task Force on standardized post-processing. J Cardiovasc Magn Reson.

[4] Chan RH, Maron BJ, Olivotto I (2014). Prognostic value of quantitative contrast-enhanced cardiovascular magnetic resonance for the evaluation of sudden death risk in patients with hypertrophic cardiomyopathy. Circulation.

[5] Marrakchi S, Kammoun I, Bennour E, Laroussi L, Kachboura S (2020). Risk stratification in hypertrophic cardiomyopathy. Herz.

[6] McDonald RJ, Levine D, Weinreb J (2018). Gadolinium retention: a research roadmap from the 2018 NIH/ACR/RSNA workshop on gadolinium chelates. Radiology.

[7] Todd DJ, Kay J (2016). Gadolinium-induced fibrosis. Annu Rev Med.

[8] Gillies RJ, Kinahan PE, Hricak H (2016). Radiomics: images are more than pictures, they are data. Radiology.

[9] Raisi-Estabragh Z, Izquierdo C, Campello VM (2020). Cardiac magnetic resonance radiomics: basic principles and clinical perspectives. Eur Heart J Cardiovasc Imaging.

[10] Cheng S, Fang M, Cui C (2018). LGE-CMR-derived texture features reflect poor prognosis in hypertrophic cardiomyopathy patients with systolic dysfunction: preliminary results. Eur Radiol.

[11] Chen BH, An DA, He J (2021). Myocardial extracellular volume fraction radiomics analysis for differentiation of reversible versus irreversible myocardial damage and prediction of left ventricular adverse remodeling after ST-elevation myocardial infarction. Eur Radiol.

[12] Neisius U, El-Rewaidy H, Nakamori S, Rodriguez J, Manning WJ, Nezafat R (2019). Radiomic analysis of myocardial native T1 imaging discriminates between hypertensive heart disease and hypertrophic cardiomyopathy. JACC Cardiovasc Imaging.

[13] Neisius U, El-Rewaidy H, Kucukseymen S (2020). Texture signatures of native myocardial T1 as novel imaging markers for identification of hypertrophic cardiomyopathy patients without scar. J Magn Reson Imaging.

[14] Schofield R, Ganeshan B, Fontana M (2019). Texture analysis of cardiovascular magnetic resonance cine images differentiates aetiologies of left ventricular hypertrophy. Clin Radiol.

[15] Speiser JL, Miller ME, Tooze J, Ip E (2019). A comparison of random forest variable selection methods for classification prediction modeling. Expert Syst Appl.

[16] O'Mahony C, Jichi F, Monserrat L (2016). Inverted U-shaped relation between the risk of sudden cardiac death and maximal left ventricular wall thickness in hypertrophic cardiomyopathy. Circ Arrhythm Electrophysiol.

[17] Authors/Task Force m, Elliott PM, Anastasakis A et al (2014). 2014 ESC guidelines on diagnosis and management of hypertrophic cardiomyopathy: the task force for the diagnosis and Management of Hypertrophic Cardiomyopathy of the European Society of Cardiology (ESC). Eur Heart J 35:2733-2779. 10.1093/eurheartj/ehu284 25173338

[18] Vigneault DM, Yang E, Jensen PJ (2019). Left ventricular strain is abnormal in preclinical and overt hypertrophic cardiomyopathy: cardiac MR feature tracking. Radiology.

[19] Xu HY, Chen J, Yang ZG (2017). Early marker of regional left ventricular deformation in patients with hypertrophic cardiomyopathy evaluated by MRI tissue tracking: the effects of myocardial hypertrophy and fibrosis. J Magn Reson Imaging.

[20] Qin L, Min J, Chen C (2021). Incremental values of T1 mapping in the prediction of sudden cardiac death risk in hypertrophic cardiomyopathy: a comparison with two guidelines. Front Cardiovasc Med.

[21] Baessler B, Mannil M, Oebel S, Maintz D, Alkadhi H, Manka R (2018). Subacute and chronic left ventricular myocardial scar: accuracy of texture analysis on nonenhanced cine MR images. Radiology.

[22] Gitto S, Cuocolo R, van Langevelde K (2022). MRI radiomics-based machine learning classification of atypical cartilaginous tumour and grade II chondrosarcoma of long bones. EBioMedicine.

[23] Jiang M, Li CL, Luo XM (2022). Radiomics model based on shear-wave elastography in the assessment of axillary lymph node status in early-stage breast cancer. Eur Radiol.

[24] Mancio J, Pashakhanloo F, El-Rewaidy H (2022). Machine learning phenotyping of scarred myocardium from cine in hypertrophic cardiomyopathy. Eur Heart J Cardiovasc Imaging.

[25] Scatteia A, Baritussio A, Bucciarelli-Ducci C (2017). Strain imaging using cardiac magnetic resonance. Heart Fail Rev.

[26] Claus P, Omar AMS, Pedrizzetti G, Sengupta PP, Nagel E (2015). Tissue tracking technology for assessing cardiac mechanics: principles, normal values, and clinical applications. JACC Cardiovasc Imaging.

[27] Qin L, Chen C, Gu S (2021). A radiomic approach to predict myocardial fibrosis on coronary CT angiography in hypertrophic cardiomyopathy. Int J Cardiol.

[28] Zhang N, Yang G, Gao Z (2019). Deep learning for diagnosis of chronic myocardial infarction on nonenhanced cardiac cine MRI. Radiology.

[29] Zhang Q, Burrage MK, Lukaschuk E (2021). Toward replacing late gadolinium enhancement with artificial intelligence virtual native enhancement for gadolinium-free cardiovascular magnetic resonance tissue characterization in hypertrophic cardiomyopathy. Circulation.

