# Supplementary material for: Identification of fibrosis in hypertrophic cardiomyopathy: a radiomic study on cardiac magnetic resonance cine imaging
Source: Eur Radiol. 2022 Nov 5;33(4):2301–11. doi: 10.1007/s00330-022-09217-0 (PMC10017609; doi:10.1007/s00330-022-09217-0)
Supplement: Supplementary file 1 — (DOCX 656 kb) [file 330_2022_9217_MOESM1_ESM.docx]

**Supplementary material**

***Supplementary Figure S1*.** The importance level of selected radiomic features from the maximum wall thickness slice (a) and entire left ventricular myocardium (b). The cluster represents similar variable importance scores.


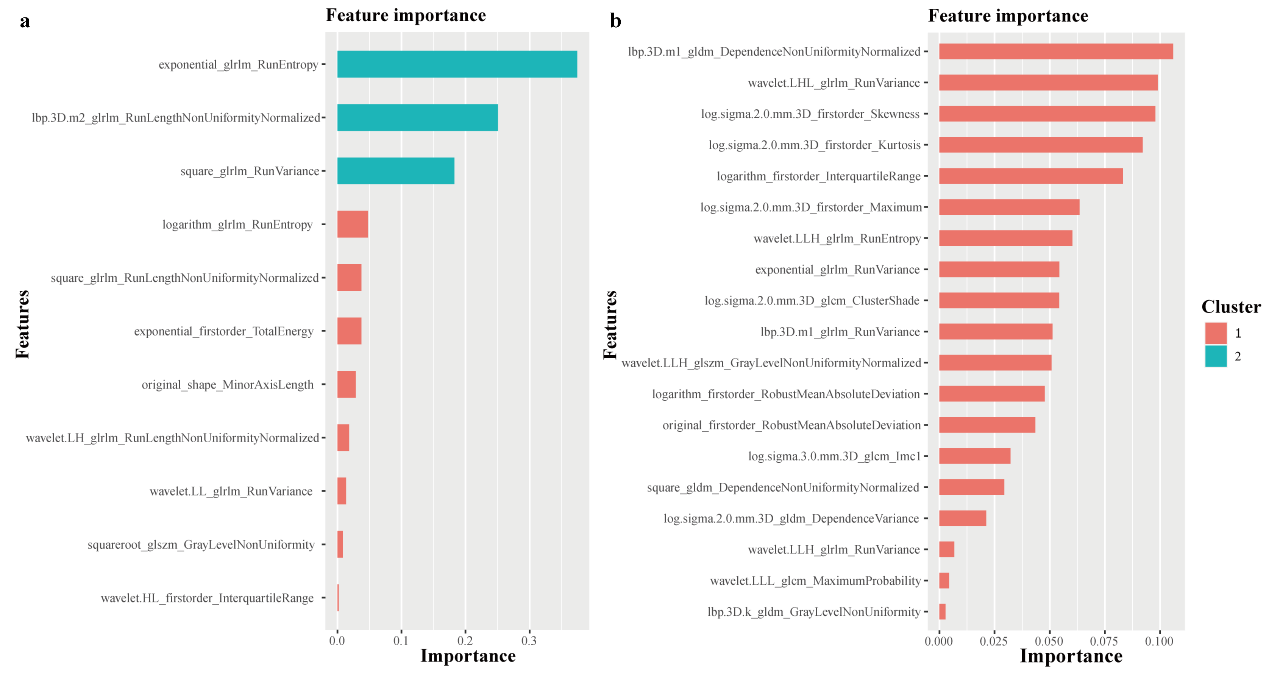


***Supplementary Figure S2*.** Boxplots of predictive score in the training (a) and test (b) sets. HCM patients with positive LGE had higher scores. R1: radiomics derived from the maximum wall thickness slice; R2: radiomics derived from the entire left ventricular myocardium. *HCM*, hypertrophic cardiomyopathy; *LGE*, late gadolinium enhancement.


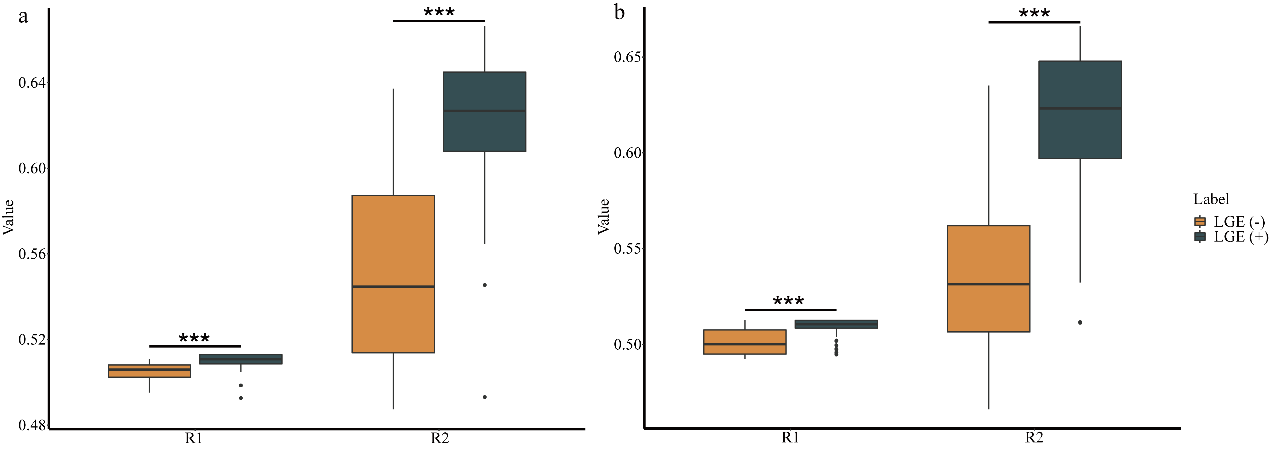


***: *P* < 0.001.

***Supplementary Figure S3.*** Examples of myocardial appearance in cine imaging by radiomic analysis to reveal fibrosis: one without fibrosis (upper row, a-d) and one with fibrosis (bottom row, e-h). Cine images (a, e) and corresponding LGE images (b, f), region of interest (red circle) for radiomic analysis (c, g), and normalized maps (d, h). HCM patient with fibrosis can be identified by radiomic analysis in cine images, showing higher myocardial signal intensity and more heterogenous myocardial texture.


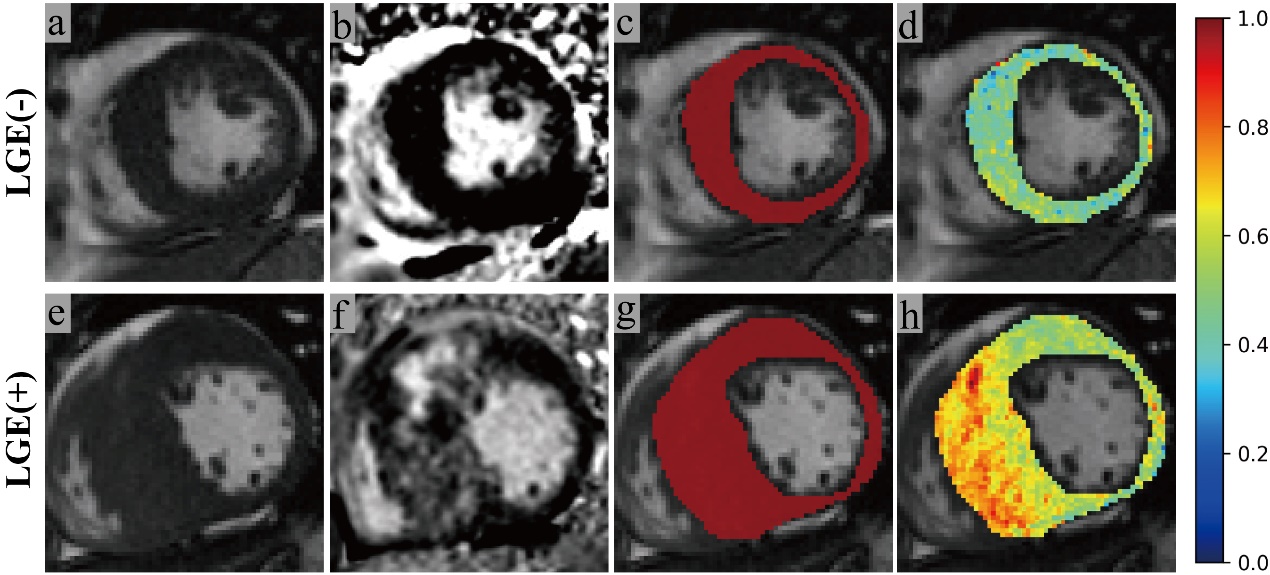


***Supplementary Table S1*：**Sequence parameters of two MR scanners

| **Parameter** | **GE** | | **Siemens** | |
| --- | --- | --- | --- | --- |
|  | **Cine** | **LGE** | **Cine** | **LGE** |
| Slice thickness/ gap (mm) | 8/ 2 | 8/ 2 | 8/ 2 | 8/ 2 |
| Repetition time (ms) | 3.5 | 6.6 | 2.64 | 2.5 |
| Echo time (ms) | 1.5 | 3.1 | 1.11 | 1.09 |
| Flip angle (degrees) | 45 | 20 | 56 | 40 |
| FOV (mm^2^) | 360×360 | 360×324 | 340×276 | 340×255 |
| Acquired matrix (pixels^2^) | 224×224 | 256×192 | 192×125 | 192×116 |
| Reconstructed matrix (pixels^2^) | 512×512 | 512×512 | 192×156 | 192×144 |
| Temporal resolution (ms) | 49 | NA | 47.52 | NA |
| Cardiac phase | 20 | NA | 25 | NA |

Abbreviations: *FOV*, field of view; *LGE*, late gadolinium enhancement

***Supplementary Table S2.*** The top radiomic features in R1, R2, and correlated interpretations in LGE(+) patients with HCM

| **Rank** | **Feature name** | **Feature Type** | **Method** | **Importance** | **Meaning in LGE(+) patients** |
| --- | --- | --- | --- | --- | --- |
| **The MWT slice** |  |  |  |  |  |
| 1 | Run Entropy | GLRLM | Exponential | 0.3743 | Random and heterogeneous  myocardial texture |
| 2 | Run Length Non-Uniformity Normalized | GLRLM | Lbp-3D-m2 | 0.2505 | Nonuniform and coarse myocardial texture |
| 3 | Run Variance | GLRLM | Square | 0.1825 | coarse myocardial texture |
| **The entire LV** |  |  |  |  |  |
| 1 | Dependence Non-Uniformity Normalized | GLDM | Lbp-3D-m1 | 0.1059 | Nonuniform and coarse myocardial texture |
| 2 | Run Variance | GLRLM | Wavelet-LHL | 0.0990 | coarse myocardial texture |
| 3 | Skewness | First order | Log-sigma-2-0-mm-3D | 0.0979 | Nonuniform myocardial signal intensity |
| 4 | Kurtosis | First order | Log-sigma-2-0-mm-3D | 0.0921 | Higher myocardial signal intensity |
| 5 | Interquartile Range | First order | Logarithm | 0.0832 | Nonuniform myocardial signal intensity |

Abbreviations: *GLDM*, Gray Level Dependence Matrix; *GLRLM*, Gray Level Run Length Matrix; *HCM*, hypertrophic cardiomyopathy; *LV*, left ventricular; *MWT*, maximum wall thickness; others, see *Supplementary Table S1*

***Supplementary Table S3*：**AUC values of R2 and I_CMR+R2_ models between different scanners

| **Model** | **Training set** | | | ***P1*** | ***P2*** | **Test set** | | | ***P3*** | ***P4*** |
| --- | --- | --- | --- | --- | --- | --- | --- | --- | --- | --- |
|  | **Mixed** | **GE** | **Siemens** |  |  | **Mixed** | **GE** | **Siemens** |  |  |
| R_2_ | 0.909 | 0.916 | 0.888 | 0.858 | 0.673 | 0.906 | 0.909 | 0.905 | 0.965 | 0.990 |
| I_CMR+R2_ | 0.930 | 0.929 | 0.920 | 0.983 | 0.802 | 0.898 | 0.909 | 0.900 | 0.856 | 0.977 |

*P1, P3*: Mixed vs GE; *P2, P4*: Mixed vs Siemens

Abbreviations: *AUC*, area under the curve
